# Changes in general and abdominal obesity in children at 4, 6 and 9 years of age and their association with other cardiometabolic risk factors

**DOI:** 10.1007/s00431-022-04802-3

**Published:** 2023-01-14

**Authors:** Honorato Ortiz-Marrón, Gloria Cabañas Pujadas, Maira Alejandra Ortiz-Pinto, Aránzazu Martín García, Carolina Matesanz Martínez, María del Castillo Antonaya Martín, Olga Cortés Rico, Iñaki Galán

**Affiliations:** 1 Department of Epidemiology, General Directorate of Public Health, Ministry of Health, C/López de Hoyos, n° 35, 1st Floor, Community of Madrid, 28006 Madrid, Spain; 2 Internal Medicine Service, Ávila Healthcare Complex, Ávila, Spain; 3 Clinical Analysis and Biochemistry Service, Puerta de Hierro University Hospital, Majadahonda, Community of Madrid, Spain; 4 Las Calesas Health Center, Eastern Area of Primary Care of Madrid, Community of Madrid, Madrid, Spain; 5 Mar Baltic Health Center, Eastern Area of Primary Care of Madrid, Community of Madrid, Madrid, Spain; 6 Canillejas Health Center, Northern Area of Primary Care of Madrid, Community of Madrid, Madrid, Spain; 7 National Center for Epidemiology, Institute of Health Carlos III, Madrid, Spain; 8 Department of Preventive Medicine and Public Health, Autonomous University of Madrid/IdiPAZ, Madrid, Spain

**Keywords:** Cardiometabolic risk, Children, Dysglycemia, Dyslipidemia, Insulin resistance, High blood pressure, Obesity

## Abstract

Temporary changes in childhood obesity and their association with cardiometabolic risk factors have been receiving increased attention. The objective of this study was to evaluate changes in general (GO) and abdominal (AO) obesity in children from 4 to 9 years of age and their associations with cardiometabolic risk factors at 9 years of age. This study includes 1344 children from the Longitudinal Childhood Obesity Study (ELOIN). Physical examinations performed at 4, 6 and 9 years of age and a blood sample was only taken at 9 years of age. Changes in obesity from 4 to 9 years of age were estimated using Body Mass Index and waist circumference. Participants were classified into four groups according to GO and AO: (1) stable without obesity (no obesity at all three measurements); (2) remitting obesity at 9 years (obesity at 4 and/or 6 years but not at 9 years); (3) incident or recurrent obesity at 9 years (obesity only at 9 years, at 4 and 9 years or at 6 and 9 years); and (4) stable or persistent with obesity (obesity at 4, 6 and 9 years). Dyslipidemia and dysglycemia were defined by the presence of at least one altered parameter of the lipid or glycemic profile. Odds ratios (OR) were estimated using logistic regression. Compared with children without GO at all ages, those with persistent GO had an OR of 3.66 (95% CI: 2.06–6.51) for dyslipidemia, 10.61 (95% CI: 5.69–19.79) for dysglycemia and 8.35 (95% CI: 4.55–15.30) for high blood pressure. The associations were fairly similar in the case of AO, with ORs of 3.52 (95% CI: 1.96–6.34), 17.15 (95% CI: 9.09–32.34) and 8.22 (95% CI: 4.46–15.15), respectively, when comparing persistent versus stable without AO. Children with incident obesity at 9 years presented a moderate cardiometabolic risk that was nevertheless higher compared to those stable without obesity, whereas those with remitting obesity did not show any significant associations.

*Conclusion*: Incident, and especially, persistent obesity, is associated with an increased cardiometabolic risk. The very early prevention of obesity, with a focus on nutrition, physical activity and sedentary behaviour, as well as tracking growth from birth to age 5, should be a priority to prevent the burden of cardiometabolic disease with consequences for adulthood.

**Table:** 

**What is Known:** *• General and abdominal obesity has been shown to be associated with other cardiometabolic risk factors such as dyslipidemia, insulin resistance and hypertension.* *• Temporary changes in obesity and their associations with cardiometabolic risk factors have not been sufficiently explored in childhood.*	
**What is New:** *• Children with incident, and especially persistent, general and/or abdominal obesity, had an increased risk of dyslipidemia, dysglycemia and high blood pressure.* *•Remitting obesity was not associated with an increased cardiometabolic risk.*

**Supplementary Information:**

The online version contains supplementary material available at 10.1007/s00431-022-04802-3.

## Introduction

Obesity is a major public health problem that affects more than 300 million children worldwide [1]. Childhood obesity has negative effects on physical and mental health [2, 3] and tends to persist in adulthood, carrying an increased risk of morbidity and mortality [4, 5]. In Europe, the prevalence of overweight and obesity during childhood is high, with large variations between regions and a higher prevalence in the Mediterranean basin [6]. In Spain, the prevalence of obesity in the population aged 6 to 9 years, using World Health Organization (WHO) criteria, was 17.3% in 2019 [7].

Moreover, cardiovascular diseases are the main cause of death in Western countries [8]; in 2018, they were the leading cause of death in Spain, accounting for 28.3% of all deaths [9]. Obesity and other cardiometabolic risk factors (CMRFs), such as high triglyceride levels, low high-density lipoprotein cholesterol (Col-HDL) levels, high blood pressure (BP) and insulin resistance, initiate atherosclerosis in childhood, which subsequently produces subclinical cardiovascular disease in adulthood [10]. Recently, a large prospective study showed that the presence of these factors in childhood is associated with fatal and nonfatal cardiovascular events in young adults [11].

General (GO) and abdominal obesity (AO) are associated with dyslipidemia, diabetes, insulin resistance, hypertension and a state of general inflammation that increase cardiovascular risk in childhood and throughout life; these relationships are well-documented in adolescents but underexplored in children, especially preschool-age children [12–15].

CMRFs typically co-occur and the presence of three or more of these risk factors is defined as metabolic syndrome [16]. However, given the intrinsic variations in age and growth during this childhood period, in children under 10 years of age, analysis of CMRFs should be calculated individually [17]. A longer duration and persistence of obesity are associated with a worse prognosis on all CMFRs [18], and the study of BMI trajectories during childhood can serve to predict future cardiometabolic risk [19, 20].

The objective of the study was to determine the association between changes in GO and AO at 4, 6 and 9 years of age and the risk of developing cardiometabolic alterations, such as dyslipidemia, dysglycemia and high blood pressure at 9 years in a prospective cohort of children representative of the population in Community of Madrid, Spain. Our hypothesis was that exposure to persistent or recurrent obesity during childhood would significantly impact on the subsequent development of cardiometabolic alterations, even during childhood and preadolescence.

## Materials and methods

### Study population

This study has a cross-sectional design in 9-year-old children. The data were extracted from the Longitudinal Childhood Obesity Study (ELOIN), a population-based prospective cohort study consisting of a baseline cohort representative of the 4-year-old population of the Community of Madrid. These data consisted of standardised anthropometric measurements, BP data and a telephone interview with parents performed at 4, 6 and 9 years as well as a blood test performed at 9 years of age. The ELOIN methodology has been previously published [21]. This study included 1344 children who had completed all three physical examinations at 4, 6 and 9 years of age and provided a blood sample at the age of 9 years. Figure S1 (Supplementary Information) shows the flowchart of participants included throughout the study period.

### Anthropometric measurements

The physical examinations were performed by paediatricians and nurses in the Sentinel Network of the 31 primary care centres that participated in this study [22]. Standardised measurements of weight, height, WC and BP were collected.

Weight measurements were performed using a digital scale, height measurements were collected with a telescopic stadiometer and the abdominal circumference was measured above the iliac crests with an approved inextensible measuring tape with a buckle. Two measurements were performed and the mean was used. Using weight and height, BMI (kg/m^2^) was estimated according to age (months) and sex. Weight status was categorised with the WHO-2007 reference tables [23] and GO was defined if the *z* score of BMI (*z*-BMI) exceeded 2 × standard deviations [24]. AO was defined according to the WC, by age and sex the using criteria of the International Diabetes Federation at the ≥ 90th percentile cut-off points proposed by Fernández et al. [25]. To classify temporary changes in GO and AO at 4, 6 and 9 years, participants were classified into four groups: (1) stable without obesity (no obesity at all three measurements); (2) remitting obesity at 9 years (obesity at 4 and/or 6 years but not at 9 years); (3) incident or recurrent obesity at 9 years (obesity only at 9 years, at 4 and 9 years or at 6 and 9 years); and (4) stable or persistent with obesity (obesity at 4, 6 and 9 years).

### Blood pressure

BP was measured at the paediatric examination using the auscultatory method from the right arm. The participants remained seated for 5 min before BP was measured. Two measurements were performed on the same day at least 2 min apart, and a third was performed if the difference between the first two measurements exceeded 4 mmHg. The mean of the measurements was used for the analyses. The BP values were standardised according to age, sex and height using the reference tables of the Fourth Report of Diagnosis, Evaluation, and Treatment of High Blood Pressure in Children and Adolescents (NHBPEP) [26].

### Biochemical parameters

The biochemical parameters were obtained from a blood sample extracted by venipuncture using Vacutainer^©^ at the health centre after fasting for 8 h.

Total cholesterol (TC) and high-density lipoprotein cholesterol (Col-HDL) were calculated by the enzymatic method of cholesterol oxidase, esterase and peroxidase; low-density lipoprotein cholesterol (Col-LDL) was calculated using the Friedewald formula [Col-LDL = (TC-Col-HDL) − (TG/5)]. Triglycerides (TG) were calculated by the lipase/glycerol kinase colorimetric method, C-reactive protein by immunoturbidimetry and glucose by the enzymatic method of glucose hexokinase coupled to glucose 6-P dehydrogenase. All determinations were performed with equipment from Siemens Healthineers.

Insulin was measured by chemiluminescence and glycated haemoglobin (HbA1c) by high-performance liquid chromatography in a TOSOH G8 analyzer. Insulin resistance (IR) was estimated using the *homeostatic model assessment of insulin resistance* (HOMA-IR), calculated as glucose (mg/dL) × insulin (µU/mL)/405.

### Definition of metabolic abnormalities

To define abnormal values of lipid and glycemic profiles, the criteria of the National Heart, Lung and Blood Institute [27] and the European Guide for Cardiovascular Prevention [28] were applied as follows:

Dyslipidemia and dysglycemia were defined by the presence of at least one altered parameter of the lipid (TC ≥ 200 mg/dL, TG ≥ 100 mg/dL, Col-HDL < 40 mg/dL or Col-LDL ≥ 130 mg/dL) or glycemic (glucose ≥ 100 mg/dL, HOMA-IR ≥ 3.16 or glycated haemoglobin ≥ 5.7%) profile, respectively.

High BP was defined by age and sex using the 90th percentile of the systolic and/or diastolic BP for children and adolescents, according to recommendations of the European Society of Hypertension [29].

#### Covariates

The sociodemographic variables were sex, age and family purchasing power; family purchasing power was estimated by the score on the *Family Affluence Scale* (FAS-II), which is a global indicator of family socioeconomic status in which family affluence is classified as low (0–3 points), medium (4–5 points) or high (6–9 points) [30].

Other covariates included as follows: dietary quality, which was evaluated according to the *Mediterranean Diet Quality Index* (Med-DQI) [31], using a semiquantitative food consumption frequency questionnaire that recorded the frequency of consumption (daily, weekly, monthly or annual) of 145 food items in the past year; and physical activity, which was determined based on the validated *Physical Activity Questionnaire-Children* (PAQ-C), on which scores range from 1 (little physical activity) to 5 (high physical activity) [32]. The information collected at 9 years of age was used for this study.

The study protocol was approved by the Ethics Committee of the Ramón y Cajal University Hospital in Madrid (CIHURC- 122/11). Written consent was obtained from the parents and/or guardians of the participants, and the data were anonymised to ensure confidentiality. The present study was conducted according to the Declaration of Helsinki and all methods were performed.

#### Statistical analysis

The descriptive statistics are expressed in percentages and means with their corresponding 95% confidence intervals (95% CIs). Analyses of variance (ANOVAs) were used to estimate the differences in means between groups, and Pearson’s chi-squared test was used to determine differences in categorical variables.

To study the association between changes in obesity and biochemical parameters, multiple linear regression models were constructed adjusted for sex, age, family purchasing power, diet quality and physical activity.

Using logistic regression, the association between changes in obesity and metabolic alterations was estimated by calculating prevalence odds ratios (ORs), adjusting for the possible confounding factors described above. Finally, through multinomial logistic regression, the association of obesity changes with combined metabolic alterations (0, 1 or 2–3 alterations of the lipid profile, glycemic profile or BP) was determined in terms of relative risk ratios (RRRs).

The threshold for statistical significance was established at *p* < 0.05.

The analyses were performed with the STATA 16.1 software (StataCorp, College Station, TX, USA).

## Results

A total of 1344 participants were included in the GO analysis, and 1324 were included in the AO analysis. Of these participants, 49% were boys, with a mean age of 9.2 years. Table 1 presents the sociodemographic characteristics of the sample. The group with stable GO was slightly older, and there were more boys than girls in the group with incident GO. Regarding GO and AO, the groups with stable and incident obesity had a lower FAS-II score (lower socioeconomic position).

**Table 1:** Characteristics of the sample according to changes in general and abdominal obesity at 4, 6 and 9 years of age

**General obesity**^**b**^	**Total**	**Stable without obesity**^**a**^	**Remitting obesity**	**Incident obesity**	**Stable with obesity**	***p* value**
	(*n* = 1344)	(*n* = 1094)	(*n* = 24)	(*n* = 171)	(*n* = 55)	
Age (months), mean (SD)	110.3 (3.9)	110.3 (3.8)	110.3 (4.0)	110.5 (4.0)	111.8 (5.0)	0.037
Sex (%)						0.003
Male	665 (49.5)	517 (47.3)	12 (50.0)	107 (62.6)	29 (52.7)	
Female	679 (50.5)	577 (52.7)	12 (50.0)	64 (37.4)	26 (47.3)	
Family purchasing power^d*^ (%)						0.002
Low	223 (17.0)	164 (15.3)	3 (13.0)	39 (23.6)	17 (33.3)	
Medium	417 (31.8)	342 (31.9)	5 (21.7)	53 (32.1)	17 (33.3)	
High	671 (51.2)	566 (52.8)	15 (65.2)	73 (44.2)	17 (33.3)	
Physical activity^e*^, mean (SD)	3.1 (0.6)	3.1 (0.6)	3.2 (0.6)	3.0 (0.6)	3.0 (0.6)	0.136
Diet quality score^f*^, mean (SD)	6.4 (1.6)	6.4 (1.6)	5.9 (1.9)	6.5 (1.7)	6.1 (1.6)	0.169

**Abdominal obesity**^**c**^	**Total**	**Stable without obesity**	**Remitting obesity**	**Incident obesity**	**Stable with obesity**	***p* value**
	(*n* = 1324)	(*n* = 1080)	(*n* = 46)	(*n* = 147)	(*n* = 51)	
Age (months), mean (SD)	110.3 (3.9)	110.3 (3.8)	110.7 (4.4)	110.5 (4.1)	111.4 (5.3)	0.182
Sex (%)						0.488
Male	655 (49.5)	535 (49.5)	21 (45.7)	78 (53.1)	21 (41.2)	
Female	669 (50.5)	545 (50.5)	25 (54.3)	69 (46.9)	30 (58.8)	
Family purchasing power^d*^ (%)						0.005
Low	222 (17.2)	164 (15.5)	10 (23.8)	32 (22.5)	16 (32.7)	
Medium	412 (31.8)	341 (32.1)	8 (19.1)	48 (33.8)	15 (30.6)	
High	660 (51.0)	556 (52.4)	24 (57.1)	62 (43.7)	18 (36.7)	
Physical activity^e*^, mean (SD)	3.1 (0.6)	3.1 (0.7)	3.1 (0.7)	3.0 (0.5)	3.0 (0.6)	0.114
Diet quality score^f*^, mean (SD)	6.4 (1.6)	6.4 (1.6)	6.3 (1.8)	6.5 (1.8)	6.1 (1.7)	0.478

*SD* standard deviation

^*^Contains missing values

^a^Stable without obesity: without obesity on all three measurements; remitting obesity: in obesity at 4 and/or 6 years old, but not at 9 years; incident obesity: in obesity only at 9 or at 4 or 6 years of age as well as 9 years of age; and stable with obesity: in obesity at 4, 6 and 9 years

^b^General obesity: body mass index (BMI) > + 2 (SD) according to the standardised tables of the WHO 2007

^c^Abdominal obesity: ≥ 90th percentile of waist circumference according to consensus of the International Diabetes Federation (IDF)

^d^Measured through the Family Affluence Scale (FAS-II)

^e^Physical Activity Questionnaire-Children (PAQ-C), scores of 1–5

^f^Mediterranean-Diet Quality Index (Med-DQI), scores from 1 to 14

Table 2 shows the means of the biochemical parameters according to the changes in obesity. In terms of the lipid profile, all GO and AO groups exhibited lower values of Col-HDL and higher values of TG and triglycerides/HDL ratio (TG/HDL ratio), than those observed in children stable without GO and stable without AO. Regarding the glycemic profile, notable differences were observed in insulin and HOMA-IR levels, which gradually widened between the groups with remitting, incident and stable obesity, reaching a maximum in the latter. Additionally, systolic and diastolic BP increased gradually in the obesity groups, reaching the highest values in the GO and stable AO groups.

**Table 2:** Cardiometabolic parameters according to changes in general and abdominal obesity at 4, 6 and 9 years of age

**General obesity**^**b**^	**Total**	**Stable without obesity**^**a**^	**Remitting obesity**	**Incident obesity**	**Stable with obesity**	***p* value**
	**Mean (SD)**					
Lipid profile (*N* = 1344)						
Total cholesterol (mg/dL)	165.0 (26.4)	165.4 (26.1)	174.1 (25.8)	162.7 (29.6)	158.8 (21.5)	0.060
HDL cholesterol (mg/dL)	60.3 (13.4)	62.0 (13.3)	57.3 (11.5)	53.5 (11.4)	50.0 (10.8)	< 0.001
Non-HDL cholesterol (mg/dL)	104.6 (24.5)	103.4 (23.9)	116.8 (23.4)	109.2 (28.4)	108.8 (19.6)	< 0.001
LDL cholesterol (mg/dL)	91.5 (23.1)	91.2 (22.7)	102.8 (21.5)	93.1 (25.7)	88.6 (19.9)	< 0.001
Triglycerides (mg/dL)	65.5 (33.4)	61.2 (27.4)	70.1 (26.6)	80.6 (43.2)	100.6 (63.2)	< 0.001
Triglycerides/HDL ratio	1.2 (0.9)	1.1 (0.6)	1.3 (0.7)	1.6 (1.1)	2.3 (2.0)	< 0.001
Glycemic profile						
Glycemia (mg/dL) (***N*** = 1343)	83.9 (7.5)	83.8 (7.6)	82.2 (6.8)	85.5 (7.1)	83.0 (7.1)	0.013
Glycated haemoglobin (%) (***N*** = 1337)	5.3 (0.3)	5.3 (0.3)	5.3 (0.3)	5.3 (0.3)	5.4 (0.3)	0.014
Insulin (µU/mL) (***N*** = 1314)	7.6 (8.1)	6.3 (5.2)	7.8 (4.1)	12.2 (9.8)	19.0 (23.3)	< 0.001
HOMA-IR^c^ (***N*** = 1314)	1.6 (2.0)	1.4 (1.5)	1.6 (0.9)	2.6 (2.3)	4.0 (5.3)	< 0.001
Blood pressure (***N*** = 1344)						
Systolic pressure (mmHg)	97.9 (11.4)	96.1 (10.4)	97.0 (10.0)	105.2 (12.2)	110.4 (11.1)	< 0.001
Diastolic pressure (mmHg)	59.0 (9.1)	57.9 (8.4)	58.9 (10.3)	63.5 (10.5)	66.6 (8.8)	< 0.001

**Abdominal obesity**^**d**^	**Total**	**Stable without obesity**^a^	**Remitting obesity**	**Incident obesity**	**Stable with obesity**	***p* value**
	Mean (SD)					
Lipid profile (***N*** = 1324)						
Total cholesterol (mg/dL)	165.0 (26.5)	165.6 (26.4)	161.1 (25.4)	164.5 (28.2)	156.4 (22.9)	0.073
HDL cholesterol (mg/dL)	60.5 (13.4)	62.1 (13.4)	57.2 (12.3)	53.9 (10.6)	47.9 (9.6)	< 0.001
Non-HDL cholesterol (mg/dL)	104.5 (24.6)	103.6 (24.4)	104.0 (20.4)	110.6 (26.9)	108.5 (21.4)	0.008
LDL cholesterol (mg/dL)	91.5 (23.1)	91.3 (23.2)	91.0 (18.7)	94.1 (24.6)	89.1 (20.0)	< 0.001
Triglycerides (mg/dL)	65.2 (32.0)	61.3 (27.3)	64.7 (23.1)	82.3 (43.7)	97.0 (52.5)	< 0.001
Triglycerides/HDL ratio	1.2 (0.8)	1.1 (0.6)	1.2 (0.6)	1.6 (1.1)	2.2 (1.6)	< 0.001
Glycemic profile						
Glycemia (mg/dL) (***N*** = 1323)	83.9 (7.5)	83.8 (7.5)	83.5 (6.1)	85.4 (7.6)	83.5 (7.1)	0.105
Glycated haemoglobin (%) (***N*** = 1317)	5.3 (0.3)	5.3 (0.3)	5.3 (0.2)	5.3 (0.3)	5.4 (0.3)	0.009
Insulin (µU/mL) (***N*** = 1295)	7.6 (8.1)	6.3 (5.2)	8.6 (5.9)	12.2 (8.3)	21.1 (24.8)	< 0.001
HOMA-IR (***N*** = 1295)	1.6 (2.0)	1.3 (1.5)	1.8 (1.3)	2.6 (2.0)	4.5 (5.6)	< 0.001
Blood pressure (***N*** = 1324)						
Systolic pressure (mmHg)	97.8 (11.3)	95.9 (10.3)	101.3 (10.2)	106.8 (11.2)	110.8 (11.6)	< 0.001
Diastolic pressure (mmHg)	59.0 (9.1)	57.8 (8.4)	60.7 (9.1)	64.6 (9.7)	66.6 (10.5)	< 0.001

*SD* standard deviation

^a^Stable without obesity: without obesity on all three measurements; remitting obesity: in obesity at 4 and/or 6 years old, but not at 9 years; incident obesity: in obesity only at 9 or at 4 or 6 years of age as well as 9 years of age; and stable with obesity: in obesity at 4, 6 and 9 years

^b^General obesity: body mass index (BMI) > + 2 (SD) according to the standardised tables of the WHO 2007

^c^Homeostatic Model Assessment-Insulin Resistance

^d^Abdominal obesity: ≥ 90th percentile of waist circumference according to consensus of the International Diabetes Federation (IDF)

Table 3 shows the association between biochemical parameters and changes in obesity, adjusting for the main covariates. Compared with children without obesity from 4 to 9 years, the incident- and stable-with-GO participants at 9 years presented lower values of Col-HDL and higher values of TG, TG/HDL ratio, insulin, HOMA-IR and BP. Similar patterns were observed with the AO coefficients. However, children with remitting obesity showed very similar values to those without obesity.

**Table 3:** Association of changes in general and abdominal obesity at 4, 6 and 9 years of age with cardiometabolic parameters at 9 years of age

**General obesity**^**b**^	**Stable without obesity**^**a**^	**Remitting obesity**	**Incident obesity**	**Stable with obesity**
	*β*^e^ (95% CI)			
Lipid profile (*N* = 1344)				
Total cholesterol (mg/dL)	(ref)	9.65 (− 1.01; 20.31)	− 1.81 (− 6.08; 2.46)	− 3.93 (− 11.12; 3.27)
HDL cholesterol (mg/dL)	(ref)	− 4.07 (− 9.25; 1.11)	− 8.38 (− 10.45; − 6.30)^**^	− 10.90 (− 14.39; − 7.41)^**^
Non-HDL cholesterol (mg/dL)	(ref)	13.72 (3.87; 23.56)^*^	6.57 (2.63; 10.51)^**^	6.97 (0.34; 13.61)^*^
LDL cholesterol (mg/dL)	(ref)	11.73 (2.45; 21.02)^*^	2.71 (− 1.01; 6.43)	− 0.66 (− 6.93; 5.60)
Triglycerides (mg/dL)	(ref)	10.18 (− 2.65; 23.02)	19.35 (14.21; 24.49)^**^	37.92 (29.26; 46.57)^**^
Triglycerides/HDL ratio	(ref)	0.27 (− 0.06; 0.58)	0.57 (0.44; 0.69)^**^	1.16 (0.95; 1.38)^**^
Glycemic profile				
Glycemia (mg/dL) (***N*** = 1343)	(ref)	− 1.46 (− 4.48; 1.56)	1.36 (0.15; 2.57)^*^	− 1.13 (− 3.17; 0.91)
Glycated haemoglobin (%) (***N*** = 1337)	(ref)	0.01 (− 0.10; 0.12)	0.03 (− 0.01; 0.07)	0.11 (0.03; 0.18)^*^
Insulin (µU/mL) (***N*** = 1S314)	(ref)	1.73 (− 1.29; 4.76)	5.78 (4.56; 7.01)^**^	12.46 (10.38; 14.54)^**^
HOMA-IR^c^ (***N*** = 1314)	(ref)	0.34 (− 0.43; 1.11)	1.26 (0.95; 1.57)^**^	2.61 (2.08; 3.14)^**^
Blood pressure (***N*** = 1344)				
Systolic pressure (mmHg)	(ref)	0.62 (− 3.71; 4.95)	8.67 (6.94; 10.40)^**^	13.51 (10.59; 16.43)^**^
Diastolic pressure (mmHg)	(ref)	1.05 (− 2.53; 4.62)	5.46 (4.03; 6.89)^**^	8.61 (6.20; 11.03)^**^

**Abdominal obesity**^**d**^	**Stable without obesity**	**Remitting obesity**	**Incident obesity**	**Stable with obesity**
	*β* (95% CI)			
Lipid profile (***N*** = 1324)				
Total cholesterol (mg/dL)	(ref)	− 3.56 (− 11.35; 4.23)	− 0.33 (− 4.89; 4.22)	− 7.59 (− 15.02; − 0.16)^*^
HDL cholesterol (mg/dL)	(ref)	− 3.84 (− 7.62; − 0.06)^*^	− 7.70 (− 9.91; − 5.49)^**^	− 13.12 (− 16.73; − 9.51)^**^
Non-HDL cholesterol (mg/dL)	(ref)	0.28 (− 6.92; 7.48)	7.37 (3.16; 11.58)^*^	5.53 (− 1.34; 12.40)
LDL cholesterol (mg/dL)	(ref)	− 0.19 (− 6.98; 6.59)	3.33 (− 0.64; 7.30)	− 1.24 (− 7.72; 5.23)
Triglycerides (mg/dL)	(ref)	2.22 (− 6.82; 11.25)	20.12 (14.84; 25.40)^**^	33.75 (25.13; 42.37)^**^
Triglycerides/HDL ratio	(ref)	0.10 (− 0.12; 0.32)	0.56 (0.43; 0.69)^**^	1.09 (0.88; 1.30)^**^
Glycemic profile				
Glycemia (mg/dL) (***N*** = 1323)	(ref)	− 0.36 (− 2.57; 1.84)	1.37 (0.07; 2.66)^*^	− 0.25 (− 2.35; 1.85)
Glycated haemoglobin (%) (***N*** = 1317)	(ref)	0.02 (− 0.06; 0.96)	0.04 (− 0.01;0.08)	0.12 (0.04; 0.19)^*^
Insulin (µU/mL) (***N*** = 1295)	(ref)	2.21 (0.01; 4.41)	5.59 (4.30; 6.88)^**^	14.45 (12.38; 16.53)^**^
HOMA-IR (***N*** = 1295)	(ref)	0.45 (− 0.11; 1.02)	1.22 (0.89; 1.55)^**^	3.07 (2.54; 3.60)^**^
Blood pressure (***N*** = 1324)				
Systolic pressure (mmHg)	(ref)	5.16 (2.08; 8.23)^*^	10.72 (8.92; 12.52)^**^	14.58 (11.64; 17.51)^**^
Diastolic pressure (mmHg)	(ref)	3.00 (0.43; 5.58)^*^	6.79 (5.29; 8.30)^**^	8.86 (6.41; 11.32)^**^

^*^p value < 0.05; ^**^p value < 0.001

^a^Stable without obesity: without obesity on all three measurements; remitting obesity: in obesity at 4 and/or 6 years old, but not at 9 years; incident obesity: in obesity only at 9 or at 4 or 6 years of age as well as 9 years of age; and stable with obesity: in obesity at 4, 6 and 9 years

^b^General obesity: body mass index (BMI) > + 2 (SD) according to the standardised tables of the WHO 2007

^c^Homeostatic Model Assessment-Insulin Resistance

^d^Abdominal obesity: ≥ 90th percentile of waist circumference according to consensus of the International Diabetes Federation (IDF)

^e^*β*: coefficient estimated by linear regression adjusted for sex, age, family purchasing power, diet quality index (Med-DQI) and physical activity (PAQ-C)

Tables 4 and 5 present the prevalence rates and ORs of cardiometabolic alterations according to changes in obesity. For both types of obesity, the prevalence and likelihood (OR) of metabolic alterations were higher in the groups with incident or persistent obesity; no significant changes were observed in children with remitting obesity. Compared with children stable without obesity from 4 to 9 years, the groups with persistent GO and AO had a greater risk of dyslipidemia (OR: 3.66, 95% CI: 2.06–6.51 and 3.52, 95% CI: 1.96–6.34, respectively), dysglycemia (OR: 10.61, 95% CI: 5.69 to 19.79 and 17.15, 95% CI: 9.09 to 32.34, respectively), HOMA-IR (OR: 24.30, 95% CI: 11.93 to 49.49 and 29.85, 95% CI: 14.71 to 60.54, respectively) and higher BP (OR: 8.35, 95% CI: 4.55 to 15.30 and 8.22, 95% CI: 4.46 to 15.15, respectively). In Table S2 (Supplementary Information), the prevalence for each of the CMRFs is presented.

**Table 4:** Prevalence of cardiometabolic risk factors according to changes in general and abdominal obesity at 4, 6 and 9 years of age

**General obesity**^**b**^	**Total**	**Stable without obesity**^**a**^	**Remitting obesity**	**Incident obesity**	**Stable with obesity**
	Prevalence (95% CI)				
Dyslipidemia^d^ (***N*** = 1344)	21.6 (19.5–23.9)	17.7 (15.6–20.1)	29.2 (14.0–51.0)	38.0 (31.0–45.6)	43.6 (31.0–57.2)
Dysglycemia^e^ (***N*** = 1344)	10.6 (9.1–12.4)	6.3 (5.0–7.9)	8.3 (1.9–29.5)	28.7 (22.3–35.9)	41.8 (29.4–55.4)
Prediabetes^f^ (***N*** = 1312)	4.4 (3.4–5.7)	3.5 (2.6–4.9)	4.1 (0.5–26.5)	8.9 (5.4–14.3)	7.5 (2.8–18.8)
HOMA–IR^g^ (***N*** = 1312)	7.5 (6.2–9.1)	3.5 (2.6–4.9)	4.1 (0.5–26.4)	22.6 (16.9–29.6)	41.5 (28.9–55.4)
High blood pressure^h^ (***N*** = 1344)	12.7 (11.0–14.6)	8.7 (7.2–10.5)	16.7 (6.1–38.3)	28.7 (22.3–35.9)	41.8 (29.4–55.4)

**Abdominal obesity**^**c**^	**Total**	**Stable without obesity**	**Remitting obesity**	**Incident obesity**	**Stable with obesity**
	Prevalence (95% CI)				
Dyslipidemia (***N*** = 1324)	21.3 (19.2–23.6)	18.0 (15.8–20.4)	23.9 (13.5–38.7)	36.7 (29.3–44.9)	45.1 (31.8–59.1)
Dysglycemia (***N*** = 1324)	10.6 (9.1–12.4)	6.3 (5.0–7.9)	13.0 (5.8–26.6)	27.2 (20.6–35.0)	52.9 (39.0–66.4)
Prediabetes (***N*** = 1293)	4.4 (3.4–5.7)	3.5 (2.5–4.8)	4.4 (1.1–16.6)	7.7 (4.3–13.5)	13.7 (6.5–26.5)
HOMA–IR (***N*** = 1293)	7.6 (6.2–9.1)	3.4 (2.5–4.7)	11.1 (4.6–24.5)	23.2 (17.0–31.1)	47.1 (33.6–61.0)
High blood pressure (***N*** = 1324)	12.7 (11.0–14.6)	9.1 (7.5–10.9)	13.0 (5.8–26.6)	28.6 (21.8–36.5)	43.1 (3.0–57.2)

^a^Stable without obesity: without obesity on all three measurements; remitting obesity: in obesity at 4 and/or 6 years old, but not at 9 years; incident obesity: in obesity only at 9 or at 4 or 6 years of age as well as 9 years of age; and stable with obesity: in obesity at 4, 6 and 9 years

^b^General obesity: body mass index (BMI) > + 2 (SD) according to the standardised tables of the WHO 2007

^c^Abdominal obesity: ≥ 90th percentile of waist circumference according to consensus of the International Diabetes Federation (IDF)

^d^At least one parameter of the following altered lipid profile: cholesterol – total cholesterol (≥ 200 mg/dL), HDL cholesterol (< 40 mg/dL), LDL cholesterol (≥ 130 mg/dL) or triglycerides (≥ 100 mg/dL)

^e^At least one parameter of the following altered glycemic profile: glycemia (≥ 100 mg/dL), HOMA-IR (Homeostatic Model Assessment-Insulin Resistance) (≥ 3.16) or glycated haemoglobin (≥ 5.7%)

^f^Prediabetes: subcategory of dysglycemia. Glycemia (≥ 100 mg/dL) and glycated haemoglobin (≥ 5.7%)

^g^Homeostatic Model Assessment-Insulin Resistance (≥ 3.16)

^h^Systolic or diastolic blood pressure above the 90th percentile

**Table 5:** Association of changes in general and abdominal obesity at 4, 6 and 9 years of age and cardiometabolic risk factors at 9 years of age

**General obesity**^**b**^	**Stable without obesity**^**a**^	**Remitting obesity**	**Incident obesity**	**Stable with obesity**
	OR^c^ (95% CI)			
Dyslipidemia^e^ (***N*** = 1344)	1 (ref)	2.22 (0.88–5.57)	2.98 (2.09–4.26)^**^	3.66 (2.06–6.51)^**^
Dysglycemia^f^ (***N*** = 1344)	1 (ref)	1.65 (0.37–7.41)	5.88 (3.83–9.02)^**^	10.61 (5.69–19.79)^**^
Prediabetes^g^* (***N*** = 1312)	1 (ref)	1.26 (0.16–9.81)	2.32 (1.23–4.39)^*^	1.86 (0.62–5.59)
HOMA–IR^g^ (***N*** = 1312)	1 (ref)	1.59 (0.20–12.93)	8.88 (5.26–14.97)^**^	24.30 (11.93–49.49)^**^
High blood pressure^i^ (***N*** = 1344)	1 (ref)	2.11 (0.69–6.44)	4.32 (2.89–6.47)^**^	8.35 (4.55–15.30)^**^

**Abdominal obesity**^**d**^	**Stable without obesity**	**Remitting obesity**	**Incident obesity**	**Stable with obesity**
	OR (95% CI)			
Dyslipidemia^e^ (***N*** = 1324)	1 (ref)	1.33 (0.65–2.71)	2.63 (1.80–3.83)^**^	3.52 (1.96–6.34)^**^
Dysglycemia^f^ (***N*** = 1324)	1 (ref)	2.23 (0.89–5.58)	5.35 (3.40–8.41)^**^	17.15 (9.09–32.34)^**^
Prediabetes^g^ (***N*** = 1293)	1 (ref)	1.18 (0.27–5.17)	2.12 (1.05–4.32)^*^	3.94 (1.61–9.60)^*^
HOMA–IR^h^ (***N*** = 1293)	1 (ref)	3.85 (1.36–10.88)	8.92 (5.20–15.30)^**^	29.85 (14.71–60.54)^**^
High blood pressure^i^ (***N*** = 1324)	1 (ref)	1.39 (0.57–3.44)	4.01 (2.63–6.12)^**^	8.22 (4.46–15.15)^**^

^*^p value < 0.05; ^**^p value < 0.001

^a^Stable without obesity: without obesity on all three measurements; remitting obesity: in obesity at 4 and/or 6 years old, but not at 9 years; incident obesity: in obesity only at 9 or at 4 or 6 years of age as well as 9 years of age; and stable with obesity: in obesity at 4, 6 and 9 years

^b^General obesity: body mass index (BMI) > + 2 (SD) according to the standardised tables of the WHO 2007

^c^Odds ratios estimated by binomial logistic regression adjusted for sex, age, family purchasing power, diet quality index (Med-DQI) and Physical Activity Questionnaire-Children (PAQ-C)

^d^Abdominal obesity: ≥ 90th percentile of waist circumference according to consensus of the International Diabetes Federation (IDF)

^e^At least one parameter of the following altered lipid profile: cholesterol – total cholesterol (≥ 200 mg/dL), HDL cholesterol (< 40 mg/dL), LDL cholesterol (≥ 130 mg/dL) or triglycerides (≥ 100 mg/dL)

^g^At least one parameter of the following altered glycemic profile: glycemia (≥ 100 mg/dL), HOMA-IR (Homeostatic Model Assessment-Insulin Resistance) (≥ 3.16) or glycated haemoglobin (≥ 5.7%)

^g^Prediabetes: subcategory of dysglycemia. Glycemia (≥ 100 mg/dL) and glycated haemoglobin (≥ 5.7%)

^h^Homeostatic Model Assessment-Insulin Resistance: subcategory of dysglycemia (HOMA-IR ≥ 3.16)

^i^Systolic or diastolic blood pressure above the 90th percentile

Table 6 shows the relationship between obesity transitions and the combined alterations in the lipid profile, glycemic profile and BP. Compared with children stable without obesity from 4 to 9 years, children with persistent and incident GO at 9 years showed RRRs of having one cardiometabolic alteration of 2.58 and 2.13, respectively, as well as RRRs of presenting two or three cardiometabolic alterations of 33.80 and 16.11, respectively. In terms of AO, the groups stable with obesity and with incident obesity at 9 years presented RRRs of having one cardiometabolic alteration of 2.73 and 2.12, respectively, as well as RRRs of showing two or three cardiometabolic alterations of 39.36 and 11.78, respectively.

**Table 6:** Association of changes in general and abdominal obesity at 4, 6 and 9 years of age and the combined cardiometabolic alterations at 9 years of age

**General obesity**^**b**^** (*n* = 1344)**	**Stable without obesity**^**a**^	**Remitting obesity**	**Incident obesity**	**Stable with obesity**
	RRR^c^ (95% CI)			
No cardiometabolic alteration^d^	-	-	-	-
One cardiometabolic alteration	1 (ref)	2.16 (0.90–5.20)^*^	2.13 (1.44–3.15)^**^	2.58 (1.26–5.29)^*^
Two or three cardiometabolic alterations	1 (ref)	4.19 (0.85–20.73)	16.11 (9.56–27.16)^**^	33.80 (15.71–72.74)^**^

**Abdominal obesity**^**e**^** (*n* = 1324)**	**Stable without obesity**	**Remitting obesity**	**Incident obesity**	**Stable with obesity**
	RRR (95% CI)			
No cardiometabolic alteration	-	-	-	-
One cardiometabolic alteration	1 (ref)	1.22 (0.62–2.41)	2.12 (1.41–3.19)^**^	2.73 (1.24–6.00)^*^
Two or three cardiometabolic alterations	1 (ref)	2.14 (0.68–6.70)	11.78 (6.94–20.01)^**^	39.36 (17.94–86.36)^**^

^*^p value < 0.05; ^**^p value < 0.001

^a^Stable without obesity: without obesity on all three measurements; remitting obesity: in obesity at 4 and/or 6 years old, but not at 9 years; incident obesity: in obesity only at 9 or at 4 or 6 years of age as well as 9 years of age; and stable with obesity: in obesity at 4, 6 and 9 years

^b^General obesity: body mass index (BMI) > + 2 (SD) according to the standardised tables of the WHO 2007

^c^RRR: relative risk ratios estimated using multinomial logistic regression models adjusted for sex, age, family purchasing power and diet quality index (Med-DQI) and physical activity (PAQ-C), for all fitness variables. Reference category: no alteration

^d^Cardiometabolic alteration: alteration of the lipid profile, glycemic profile or blood pressure

^e^Abdominal obesity: ≥ 90th percentile of waist circumference according to consensus of the International Diabetes Federation (IDF)

Tables S3 and S4 present the association of GO and AO with CMRFs at 9 years of age. The likelihood of CMRFs, in terms of ORs, was greater in children with both GO and AO than those with GO or AO only.

## Discussion

This study evaluated the association between changes in GO and AO from 4 to 9 years of age and CMRFs at 9 years of age in a population-based child cohort. The results demonstrate changes in cardiometabolic parameters in children with incident obesity and especially those with persistent obesity; these changes were characterised by an increased risk of dyslipidemia, insulin resistance and altered BP, highlighting the strength of the association with the combined CMRFs. In contrast, children with remitting obesity at some point between the ages of 4 and 6 years, but not at 9 years old did not have an excess risk on these factors.

Our results are in line with most previous studies [3, 13–15]. Childhood obesity is related to a moderate increase in TC and Col-LDL, a moderate-to-severe increase in TG and TG/HDL ratio, and a reduction in Col-HDL [33, 34]. Our study observed an increase in TG and TG/HDL ratio and a reduction in Col-HDL in both GO and AO, especially in those participants stable with obesity.

Childhood obesity does not always influence blood glucose levels. The initial alterations of blood glucose homeostasis are observed in insulin levels and IR, which is consistent with the findings of the current study. Such outcomes are of greater magnitude in the stable-with-obesity group. In addition, as noted in our study, higher levels of diastolic BP and especially systolic BP have been consistently reported to accompany excess weight and AO [35–37].

As early as 2004, Weiss et al. [16] found that children and adolescents with obesity had a greater number of altered CMRFs. Our data shows that children with GO or AO had a very high risk of having two or three cardiometabolic alterations. This early aggregation of altered CMRFs exerts a combined effect of large magnitude [38] on health in adulthood. Thus, early detection is critical for the prevention of future cardiovascular diseases and diabetes [11].

Our work utilised a classification that allows the identification of natural changes in the state of childhood obesity, indicating a strong association between GO and stable or persistent AO in children. Similarly to other studies, these results show that a longer duration of exposure to obesity in childhood, regardless of its severity, has a greater effect on CMRFs [18, 39]. To verify these results, we analysed obesity at 9 years of age with a cross-sectional design, taking into account the exposure (Tables S3 and S4). Our results confirm that the risk of having CMRF alterations is greater among children stable with obesity between ages of 4 and 9 years than among those with obesity only at 9 years of age. These findings have important implications and consequences for public health given that obesity tends to persist once is established [40, 41]. Therefore, establishing interventions for the prevention and management of obesity in very young children is crucial to reduce the cardiometabolic risk to minimum levels, with an emphasis on the benefits of maintaining normal weight from early ages. We also emphasise that the cardiometabolic parameters of children with reduced obesity were similar to those who had never been at obesity, although the small sample size of this category limits the accuracy of these results. However, this finding is consistent with studies that showed that the cardiometabolic alterations are reversed within a short period in children with obesity who return to normal weight [42, 43].

A recent study similar to ours was conducted with children and 2 years of follow-up. These authors observed an increase in metabolic alterations in children with stable and incident obesity compared with those of children stable without obesity, although these alterations were of a lower magnitude than those estimated in our study [44]. Children with remitting obesity had a comparable number of CMRFs as stable children without obesity, except for alterations in BP. Recently, Ortiz-Pinto et al. [36] reported that children with obesity at 4 years of age and high BP exhibited normalised BP when they returned to normal weight at 6 years of age. In a recent article, Norris et al. found that a longer duration of obesity was associated with worse profiles for all cardiometabolic disease risk factors, and the strength of evidence persisted even after adjusting for obesity severity. The obesity epidemic is characterised by a trend towards an earlier onset and consequently greater lifetime exposure. Therefore, health policy recommendations must aim at preventing the early onset obesity, therefore reducing lifetime exposure to obesity [18].

Another aspect to highlight is the difference in the associations of GO and AO with metabolic alterations. In our study, these differences were very small regarding lipid metabolism and BP. However, children with stable AO had a higher risk of dysglycemia than children with stable GO (OR: 17.15 vs. 10.61), especially due to increased IR. Recently, Song et al. [45] reported that children with AO exhibited greater glycemic alterations than those with GO, and Niu et al. [46] showed that children with obesity, especially AO, have altered IR markers.

Finally, we found that children with both GO and AO have a higher risk of dyslipidemia, dysglycemia and altered BP. Although BMI is viewed as the most appropriate measure of obesity due to the ease of obtaining its values and its high correlation with WC [47], we believe that the joint use of BMI and WC facilitates the identification of children with higher baseline cardiometabolic risk.

The limitations and strengths of our study should be considered for the accurate interpretation of the results. First, losses to follow-up were high because many parents did not authorise blood extraction. However, children who participated in the three follow-up measurements had sociodemographic characteristics similar to those of the baseline cohort (as seen in Table S1 in the Supplementary Information). Second, this cohort lacked data on indicators for changes in obesity between birth and 4 years; therefore, the trajectories of GO and AO are incomplete. Third, the data used to define high BP consisted of one measurement instead of, on three measurements on different days as recommended by the European Society of Hypertension [29]. In addition, BP measurement in the paediatric population is not exempt from being affected by white coat hypertension, a bias that must be taken into consideration [48]. However, there is limited evidence that this bias is differential in individuals with or without obesity. Fourth, although our regression models included the main covariates, residual confounders cannot be ruled out. Finally, the BMI and WC used to define GO and AO are only indirect measures of body fat and can lead to erroneous classifications, especially in children with a BMI close to the cut-off point for obesity [49]. Fifth, the lack of vitamin D measurement could be acknowledged among limitations due to the positive association between vitamin D deficiency, childhood obesity and other CMRFs [50]. Additionally, the results of the present study can only be extrapolated to the population of Western countries, as the target population of this study was from a European Mediterranean country. Finally, blood sampling was only performed at 9 years of age, which must be pointed out since the lack of blood samples at the ages of 4 and 6 years did not allow the analysis of CMRFs changes over time.

Regarding the strengths of this study, three measures of obesity indicators were included, facilitating detailed longitudinal interpretation of exposure to obesity. In addition, the main confounding variables were considered, including diet quality and physical activity. Finally, the anthropometric measurements were based on objective and standardised criteria and entail lower validity errors than those self-reported or reported by the parents [51].

## Conclusions

Obesity, especially persistent obesity during childhood, shows a strong positive correlation with the presence of dyslipidemia, IR, altered BP and numerous metabolic alterations. From a public health perspective, the prevention and control of obesity in early childhood is a priority and can be accomplished by screening for threshold values of BMI and WC. Additionally, for children found to be at risk of obesity, biochemical parameters of cardiometabolic risk should be evaluated.

## Supplementary Information

Below is the link to the electronic supplementary material.

Supplementary file1 (DOCX 27 KB)

Supplementary file2 (DOCX 15 KB)

Supplementary file3 (DOCX 17 KB)

Supplementary file4 (DOCX 15 KB)

Supplementary file5 (DOCX 15 KB)

## Data Availability

Available from the corresponding author upon request.

## Abbreviations

AO: Abdominal obesity
BMI: Body mass index
BP: Blood pressure
CI: Confidence interval
Col-HDL: High-density lipoprotein cholesterol
Col-LDL: Low-density lipoprotein cholesterol
ELOIN: Longitudinal Childhood Obesity Study
GO: General obesity
HbA1c: Glycated haemoglobin
HOMA-IR: Homeostatic Model Assessment-Insulin Resistance
OR: Odds ratio
SD: Standard deviation
TC: Total cholesterol
TG: Triglycerides
WC: Waist circumference
WHO: World Health Organization
