# Supplementary material for: Changes in general and abdominal obesity in children at 4, 6 and 9 years of age and their association with other cardiometabolic risk factors
Source: Eur J Pediatr. 2023 Jan 14;182(3):1329–40. doi: 10.1007/s00431-022-04802-3 (PMC10023764; doi:10.1007/s00431-022-04802-3)
Supplement: Supplementary file 1 — Supplementary file1 (DOCX 27 KB) [file 431_2022_4802_MOESM1_ESM.docx]

**Figure S1. Flowchart of participants enrolment**

4 years
T_0_

6 years
T_1_

Participants 6 years-old
(n=2,436)

Participants 9 years-old
(n=2,018)

9 years
T_2_

Participants 4 years-old
(n=3,323)

Blood sample
(n=1,958)

**STUDY POPULATION**

Physical examination (T_0_, T_1,_ and T_2_) and blood sample (T_2_)

(n=1,344)

**Author:** Honorato Ortiz Marrón et al. Department of Epidemiology, General Directorate of Public Health. Madrid, Spain

**Journal:** European Journal of Pediatrics
