# Supplementary material for: Changes in general and abdominal obesity in children at 4, 6 and 9 years of age and their association with other cardiometabolic risk factors
Source: Eur J Pediatr. 2023 Jan 14;182(3):1329–40. doi: 10.1007/s00431-022-04802-3 (PMC10023764; doi:10.1007/s00431-022-04802-3)
Supplement: Supplementary file 2 — Supplementary file2 (DOCX 15 KB) [file 431_2022_4802_MOESM2_ESM.docx]

**Table S1. Characteristics of original cohort children and the study sample.**

|  | **Original cohort** | | | **Study sample** | | | |  |
| --- | --- | --- | --- | --- | --- | --- | --- | --- |
|  | **N** | **Mean (SD) / %** | **95% CI** | | **N** | **Mean (SD) / %** | **95% CI** | |
| **Age (months)** | 3,223 | 48.6 (1.9) | (48.5–48.6) | | 1,344 | 48.5 (1.8) | (47.4–49.6) | |
| **Sex** |  |  |  | |  |  |  | |
| Boy | 1,691 | 50.9 | (49.2–52.6) | | 944 | 49.5 | (46.8–52.1) | |
| Girl | 1,632 | 49.1 | (47.4–50.8) | | 958 | 50.5 | (47.8–53.2) | |
| **Household affluence*** |  |  |  | |  |  |  | |
| Low | 512 | 18.9 | (17.5–20.4) | | 182 | 15,2 | (13.3–17.4) | |
| Medium | 831 | 30.5 | (28.8–32.3) | | 389 | 32,5 | (29.9–35.2) | |
| High | 1,375 | 50.6 | (48.7–52.5) | | 629 | 52,3 | (49.5–55.1) | |
| **General obesity** |  |  |  | |  |  |  | |
| No | 3,108 | 93.5 | (92.6–94.3) | | 1,269 | 94.4 | (93.0–95.5) | |
| Yes | 215 | 6.5 | (5.7–7.3) | | 75 | 5.6 | (4.5–6.9) | |
| **Abdominal obesity*** |  |  |  | |  |  |  | |
| No | 3,020 | 92.2 | (90.9–92.9) | | 1,240 | 92.7 | (91.1–93.9) | |
| Yes | 257 | 7.8 | (7.1–9.1) | | 98 | 7.3 | (6.0–8.8) | |
| SD: standard deviation. * Variables with missing values. | | | | | | | | |

**Author:** Honorato Ortiz Marrón et al. Department of Epidemiology, General Directorate of Public Health. Madrid, Spain

**Journal:** European Journal of Pediatrics
