# Supplementary material for: Changes in general and abdominal obesity in children at 4, 6 and 9 years of age and their association with other cardiometabolic risk factors
Source: Eur J Pediatr. 2023 Jan 14;182(3):1329–40. doi: 10.1007/s00431-022-04802-3 (PMC10023764; doi:10.1007/s00431-022-04802-3)
Supplement: Supplementary file 3 — Supplementary file3 (DOCX 17 KB) [file 431_2022_4802_MOESM3_ESM.docx]

**Table S2.** Prevalence of cardiometabolic risk factors according to changes in general and abdominal obesity at 4, 6 and 9 years of age.

| General obesity^b^ | Total | Stable without obesity ^a^ | Remitting obesity | Incident obesity | Stable with obesity | p value |
| --- | --- | --- | --- | --- | --- | --- |
|  | Prevalence (CI 95%) | | | | |  |
| Lipid profile (N=1,344) |  |  |  |  |  |  |
| High total cholesterol (mg/dL) | 9.2 (7.7–10.8) | 8.8 (7.2–10.6) | 16.7 (6.1–38.3) | 11.7 (7.6–17.5) | 5.5 (1.7–15.9) | 0.256 |
| Low HDL cholesterol (mg/dL) | 3.9 (3.0–5.1) | 2.7 (1.8–3.8) | 4.2 (0.5–26.5) | 8.2 (4.9–13.4) | 16.4 (8.6–28.9) | <0.001 |
| Non-HDL cholesterol (mg/dL) | 5.2 (4.1–6.5) | 4.6 (3.5–6.0) | 12.5 (3.8–33.9) | 8.8 (5.3–14.1) | 3.6 (0.9–13.8) | 0.043 |
| High LDL cholesterol (mg/dL) | 5.0 (3.9–6.3) | 5.0 (3.9–6.5) | 8.3 (1.9–29.5) | 5.8 (3.2–10.6) | - | 0.293 |
| High triglycerides (mg/dL) | 10.9 (9.3–12.6) | 7.1 (5.7–8.8) | 16.7 (6.1–38.3) | 26.9 (20.7–34.1) | 32.7 (21.5–46.4) | <0.001 |
| Glycemic profile |  |  |  |  |  |  |
| Altered baseline blood glucose (N = 1,244) | 1.5 (1.0–2.3) | 1.3 (0.8–2.2) | 0 | 3.5 (1.6–7.6) | 0 | 0.099 |
| High glycated hemoglobin (N = 1,282) | 3.3 (2.4–4.4) | 2.6 (1.8–3.7) | 4.3 (0.5–27.5) | 6.2 (3.3–11.1) | 8.0 (3.0–19.9) | 0.023 |
| High insulin (µU/ml) (N=1,314) | 7.4 (6.1–8.9) | 3.5 (2.5–4.7) | 4.2 (0.5–26.5) | 21.4 (15.8–28.3) | 43.4 (30.5–57.2) | <0.001 |
| High HOMA–IR^c^ (N=1,314) | 7.5 (6.2–9.1) | 3.6 (2.6–4.8) | 4.2 (0.5–26.5) | 22.6 (16.9–29.6) | 41.5 (28.9–55.4) | <0.001 |
| Blood pressure |  |  |  |  |  |  |
| High blood pressure (N = 1,344) | 12.7 (11.0–14.6) | 8.7 (7.2–10.5) | 16.7 (6.1–38.3) | 28.7 (22.3–35.9) | 41.8 (29.4–55.4) | <0.001 |
| Abdominal obesity^d^ | **Total** | **Stable without obesity** | **Remitting obesity** | **Incident obesity** | **Stable with obesity** | **p value** |
|  | Prevalence (CI 95%) | | | | |  |
| Lipid profile (N=1,324) |  |  |  |  |  |  |
| High total cholesterol | 9.2 (7.8–10.9) | 9.2 (7.6–11.0) | 10.9 (4.5–24.0) | 10.9 (6.7–17.1) | 3.9 (0.9–14.8) | 0.503 |
| Low HDL cholesterol | 3.9 (2.9–5.0) | 2.6 (1.8–3.7) | 4.3 (1.0–16.3) | 8.2 (4.7–13.9) | 3.9 (1.0–14.8) | <0.001 |
| Non-HDL cholesterol | 5.2 (4.1–6.5) | 4.6 (3.5–6.1) | 2.2 (0.3–14.6) | 10.9 (6.7–17.1) | 3.9 (0.9–14.8) | 0.010 |
| High LDL cholesterol | 4.9 (3.9–6.2) | 5.0 (3.8–6.5) | 2.2 (0.3–14.6) | 6.8 (3.7–12.2) | 0 | 0.211 |
| High triglycerides | 10.7 (9.2–12.5) | 7.4 (6.0–9.1) | 8.7 (3.2–21.5) | 26.5 (20.0–34.3) | 37.3 (24.9–51.5) | <0.001 |
| Glycemic profile |  |  |  |  |  |  |
| Altered baseline blood glucose (N = 1,323) | 1.5 (1.0–2.3) | 1.3 (0.8–2.2) | 0 | 4.1 (1.8–8.9) | 0 | 0.039 |
| High glycated hemoglobin (N = 1,363) | 3.2 (2.4–4.4) | 2.5 (1.7–3.7) | 4.3 (1.0–16.3) | 4.4 (2.0–9.5) | 14.6 (7.0–28.0) | <0.001 |
| High insulin (N=1,295) | 7.4 (6.1–9.0) | 3.2 (2.3–4.5) | 11.1 (4.6–24.5) | 21.7 (15.6–29.2) | 51.0 (37.2–64.6) | <0.001 |
| High HOMA–IR (N=1,295) | 7.6 (6.2–9.1) | 3.4 (2.5–4.7) | 11.1 (4.6–24.5) | 23.1 (16.8–30.8) | 47.1 (33.6–61.0) | <0.001 |
| Blood pressure |  |  |  |  |  |  |
| High blood pressure (N = 1,324) | 12.7 (11.0–14.6) | 9.1 (7.5–10.9) | 13.0 (5.8–26.6) | 28.6 (21.8–36.5) | 43.1 (30.1–57.2) | <0.001 |
| ^a^ Stable without obesity: without obesity on all three measurements; remitting obesity: in obesity at 4 and/or 6 years old, but not at 9 years; Incident obesity: in obesity only at 9 or at 4 or 6 years of age as well as 9 years of age; and Stable with obesity: in obesity at 4, 6 and 9 years.  ^b^ General obesity: body mass index (BMI) > +2 (SD) according to the standardized tables of the WHO 2007.  ^c^ HOMA-IR: Homeostatic Model Assessment - Insulin Resistance.  ^d^ Abdominal obesity: ≥90^th^ percentile of waist circumference according to consensus of the International Diabetes Federation (IDF). | | | | | | |

**Author:** Honorato Ortiz Marrón et al. Department of Epidemiology, General Directorate of Public Health. Madrid, Spain

**Journal:** European Journal of Pediatrics
