# Supplementary material for: Changes in general and abdominal obesity in children at 4, 6 and 9 years of age and their association with other cardiometabolic risk factors
Source: Eur J Pediatr. 2023 Jan 14;182(3):1329–40. doi: 10.1007/s00431-022-04802-3 (PMC10023764; doi:10.1007/s00431-022-04802-3)
Supplement: Supplementary file 4 — Supplementary file4 (DOCX 15 KB) [file 431_2022_4802_MOESM4_ESM.docx]

**Table S3.** Association of general and abdominal obesity, isolated or combined, with cardiometabolic risk factors at 9 years of age.

|  | No general or abdominal obesity | General obesity, no abdominal obesity^a^ | Abdominal obesity, no general obesity | General and abdominal obesity |
| --- | --- | --- | --- | --- |
|  | OR (95% CI)^b^ | | | |
| Lipid profile (N=1,950) |  |  |  |  |
| High total cholesterol | 1 (ref) | 1.37 (0.68–2.73) | 1.00 (0.39–2.56) | 0.95 (0.57–1.58) |
| Low HDL cholesterol | 1 (ref) | 3.30 (1.40–7.75)^†^ | 3.21 (1.20–8.56)^†^ | 4.96 (2.96–8.31)^††^ |
| Non-HDL cholesterol | 1 (ref) | 1.82 (0.76–4.39) | 2.48 (1.01–6.06)^†^ | 2.22 (1.30–3.78)^†^ |
| High LDL cholesterol | 1 (ref) | 1.06 (0.37–3.00) | 1.45 (0.51–4.16) | 1.33 (0.73–2.43) |
| High triglycerides | 1 (ref) | 3.63 (2.05–6.42)^††^ | 5.02 (2.71–9.30)^††^ | 6.47 (4.54–9.22)^††^ |
| Glycemic profile |  |  |  |  |
| Altered baseline blood glucose (N = 1,804) | 1 (ref) | - | - | 2.19 (0.90–5.34) |
| High glycated hemoglobin (N = 1,869) | 1 (ref) | 0.73 (0.17–3.11) | 1.51 (0.35–6.53) | 3.03 (1.65–5.57)^††^ |
| High insulin (N=1,914) | 1 (ref) | 3.54 (1.65–7.60)^†^ | 4.98 (2.26–10.94)^††^ | 15.61 (10.38–23.49)^††^ |
| High HOMA–IR^c^ (N=1,911) | 1 (ref) | 3.20 (1.50–6.84)^†^ | 3.21 (1.50–7.55)^†^ | 15.94 (10.67–23.81)^††^ |
| Blood pressure (N=1,950) |  |  |  |  |
| High blood pressure | 1 (ref) | 2.46 (1.36–4.46)^†^ | 2.08 (0.99–4.38) | 5.74 (4.07–8.10)^††^ |
| ^a^ General obesity: body mass index (BMI) > +2 (SD) according to the standardized tables of the WHO 2007.  obesity: ≥90^th^ percentile of waist circumference according to the consensus of the International Diabetes Federation (IDF).  ^b^ Odds ratios estimated by binomial logistic regression adjusted for sex, age, family purchasing power, diet quality index (Med-DQI) and physical activity (PAQ-C).  ^c^ Homeostatic Model Assessment - Insulin Resistance.  ^†^ *p value* <0.05; ^††^ *p value* <0.001. | | | | |

**Author:** Honorato Ortiz Marrón et al. Department of Epidemiology, General Directorate of Public Health. Madrid, Spain

**Journal:** European Journal of Pediatrics
