# Supplementary material for: Changes in general and abdominal obesity in children at 4, 6 and 9 years of age and their association with other cardiometabolic risk factors
Source: Eur J Pediatr. 2023 Jan 14;182(3):1329–40. doi: 10.1007/s00431-022-04802-3 (PMC10023764; doi:10.1007/s00431-022-04802-3)
Supplement: Supplementary file 5 — Supplementary file5 (DOCX 15 KB) [file 431_2022_4802_MOESM5_ESM.docx]

**Table S4.** Association of general and abdominal obesity, isolated or combined, with cardiometabolic risk factors at 9 years of age.

|  | No obesity | General obesity ^a^ | Abdominal obesity ^b^ | General and abdominal obesity | |
| --- | --- | --- | --- | --- | --- |
|  | OR^c^ (95% CI) | | | |  |
| Dyslipidemia^d^ (N=1,950) | 1 (ref) | 2.55 (1.60–4.07)^††^ | 2.46 (1.41–4.31)^†^ | 3.87 (2.87–5.22)^††^ | |
| Dysglycemia^e^ (N=1,950) | 1 (ref) | 1.92 (0.98–3.76) | 2.11 (0.98–4.70) | 9.72 (6.90–13.69)^††^ | |
| Prediabetes^f^ (N=1,904) | 1 (ref) | 0.52 (0.12–2.20) | 0.99 (0.23–4.21) | 2.76 (1.63–4.64)^††^ | |
| HOMA–IR^g^ (N=1,904) | 1 (ref) | 3.19 (1.49–6.83)^†^ | 3.21 (1.36–7.54)^†^ | 15.92 (10.66–23.78)^††^ | |
| High blood pressure^h^ (N = 1,950) | 1 (ref) | 2.46 (1.36–4.46)^†^ | 2.08 (0.99–4.38) | 5.74 (4.07–8.10)^††^ | |
| ^a^ General obesity: body mass index (BMI) > +2 (SD) according to the standardized tables of the WHO 2007.  ^b^ Abdominal obesity: ≥90^th^ percentile of waist circumference according to the consensus of the International Diabetes Federation (IDF).  ^c^ Odds ratios estimated by binomial logistic regression adjusted for sex, age, family purchasing power, diet quality index (Med-DQI) and physical activity (PAQ-C).  ^d^ At least one parameter of the following altered lipid profile: total cholesterol (≥200 mg/dL), HDL cholesterol (<40 mg/dL), LDL cholesterol (≥130 mg/dL) and triglycerides (≥100 mg/dL).  ^e^ At least one parameter of the following altered glycemic profile: glycemia (≥100 mg/dL), HOMA-IR (≥3.16) or glycated hemoglobin (≥5.7%).  ^f^ Prediabetes: subcategory of dysglycemia. Glycemia (≥100 mg/dL) and glycated hemoglobin (≥5.7%).  ^g^ HOMA-IR: subcategory of dysglycemia. HOMA–IR ≥3.16.  ^h^ Systolic or diastolic blood pressure above the 90^th^ percentile.  ^†^ *p value* <0.05; ^††^ *p value* <0.001. | | | | |  |

**Author:** Honorato Ortiz Marrón et al. Department of Epidemiology, General Directorate of Public Health. Madrid, Spain

**Journal:** European Journal of Pediatrics
